# Services to improve outcomes in severe mental disorders

**DOI:** 10.1007/s40211-025-00539-1

**Published:** 2025-08-27

**Authors:** Merete Nordentoft

**Affiliations:** 1 Department of Clinical Medicine, Faculty of Health and Medical Sciences, University of Copenhagen, Copenhagen, Denmark; 2 CORE – Copenhagen Research Center for Mental Health, Mental Health Center Copenhagen, Mental Health Services in the Capital Region of Denmark, Copenhagen, Denmark

**Keywords:** Psychosis, Schizophrenia, Early intervention services, Assertive community treatment, Homelessness, Psychose, Schizophrenie, Frühinterventionen, „Assertive community treatment“, Prognose, Virtuelle Realität

## Abstract

Severe mental disorders, particularly schizophrenia and related disorders, remain among the most disabling and costly health conditions worldwide. Despite advances in pharmacological and psychosocial treatments, recovery rates remain low. Early intervention has emerged as a key strategy to improve outcomes. Reducing the duration of untreated psychosis (DUP) and providing specialized early intervention services—such as Denmark’s OPUS, the UK’s LEO, and the US Recovery After an Initial Schizophrenia Episode (RAISE) trial—have shown significant benefits in symptom reduction, functioning, and cost-effectiveness. Assertive Community Treatment (ACT), Individual Placement and Support (IPS), and Housing First programs further enhance recovery for individuals with complex needs, including comorbid substance use and homelessness. Innovative therapies, such as avatar-based treatment for persistent hallucinations, show promise in treatment-resistant cases. Children of parents with severe mental illness are at elevated risk and offer a unique opportunity for prevention. The Danish High Risk and Resilience Study (VIA 7), which follows high-risk children from age 7 to 19, exemplifies how longitudinal research can identify early modifiable risk factors and inform timely interventions. To improve long-term outcomes, services must be coordinated, person-centered, and recovery-oriented—delivering care that is accessible, humane, and tailored to the individual’s stage of illness and life circumstances.

## Abstract (de)

Schwere psychische Erkrankungen, insbesondere Schizophrenie und assoziierte Erkrankungen, gehören nach wie vor zu den am stärksten beeinträchtigenden und finanziell aufwendigsten Erkrankungen weltweit. Trotz der erreichten Fortschritte in pharmakologischen und psychosozialen Behandlungen sind die Heilungsraten nach wie vor niedrig. Früh zu intervenieren hat als wesentliche Strategie zur Verbesserung von Verläufen herausgestellt. Die DUP(„duration of untreated psychosis“)-Dauer zu reduzieren und spezialisierte Frühinterventionsangebote zur Verfügung zu stellen – wie etwa OPUS in Dänemark, LEO im Vereinigten Königreich und NAVIGATE im Rahmen der Recovery After an Initial Schizophrenia Episode(RAISE)-Studie in den USA – hat erhebliche Vorteile in Bezug auf Symptomreduktion, Funktionsgrad und Kosteneffizienz gezeigt. „Assertive community treatment“ (ACT), „individual placement and support“ (IPS) und Housing-First-Programme fördern die Verbesserung der Symptomatik von Personen mit komplexen Bedarfen, etwa bei komorbidem Substanzgebrauch oder Wohnungslosigkeit. Innovative Behandlungen, wie die Avatar-basierte Therapie bei persistierenden Halluzinationen, zeigen vielversprechende Ergebnisse. Kinder von Eltern mit schweren psychischen Erkrankungen sind einem erhöhten Risiko ausgesetzt und bieten eine besondere Chance für die Prävention. Die dänische „High Risk and Resilience Study“ (VIA 7), die Hochrisikokinder zwischen 7 und 19 Jahren begleitet, zeigt, wie sich durch longitudinale Forschung modifizierbare Risikofaktoren erkennen lassen können und dies eine Grundlage für zeitgerechte Interventionen darstellen kann. Um Langzeitverläufe zu optimieren, müssen psychiatrische Dienste koordiniert, personenzentriert und auf Heilung ausgerichtet sein – sie müssen Versorgung bieten, die zugänglich, empathisch und bedarfsgerecht ist, angepasst an das Krankheitsstadium und die Lebensumstände jedes Einzelnen.

## Introduction

Severe mental disorders, particularly schizophrenia and related psychoses, are among the most disabling illnesses globally. They are associated with profound personal suffering, high societal costs, and dramatically shortened life expectancy—often by 10–15 years [1]. Despite pharmacological and psychosocial advancements, for a large group, outcomes have remained poor for decades. In a meta-analysis from 2023, covering 26 study samples (*n* = 3877), a recovery rate of 20.8% was found [2].

## Shortening the duration of untreated psychosis

The duration of this period, from the onset of psychotic symptoms and initiation of treatment, termed duration of untreated psychosis (DUP), has been shown to be associated with later outcome for patients suffering from schizophrenia. One intervention study, the Treatment and Intervention in Psychosis (TIPS) study, has successfully reduced the DUP and showed effects on functional outcomes 10 years later [3], indicating that the DUP could be one of the few malleable risk factors for the outcome in schizophrenia which, if reduced, could improve outcomes.

## Early Intervention Services

The critical period hypothesis posits that early years after onset of psychosis are decisive for long-term outcomes. Intervening early during this period—before disability becomes entrenched—can reduce the severity and chronicity of illness. This rationale underpins the development of specialized Early Intervention Services for first-episode psychosis, which have emerged as one of the most significant innovations in psychiatric care in recent decades. Evidence now suggests that timely, intensive, and coordinated care can meaningfully improve clinical and functional outcomes.

Early intervention services deliver

- Low caseload case management,
- Assertive outreach,
- Psychoeducation and family involvement,
- Social skills training, and
- Evidence-based pharmacotherapy.

Multidisciplinary teams provide these services in a cohesive, integrated format, leading to greater effectiveness than fragmented care.

A 2018 meta-analysis [4] including 10 randomized controlled trials (RCTs) and 2176 patients showed that Early Intervention Services were associated with superior outcomes compared with treatment as usual regarding all analysed outcome including hospitalization risk, bed-days, symptoms, global functioning, total symptom severity, psychotic and negative symptoms, and recovery.

*The Danish OPUS I trial* demonstrated superior outcomes over 2 years in symptomatology, substance use, treatment adherence, use of bed days, and social functioning compared to treatment as usual [5].

Moreover, over 5 years, OPUS treatment was more effective and less costly, largely due to reductions in inpatient care and supported housing utilization [6]. Since 2003, Denmark has scaled the OPUS model nationally. In a register-based study, it was examined whether treatment effects were sustainable after OPUS had been implemented as standard care. The study was based on 5‑year follow-up of 3328 patients, whereby patients treated in OPUS had fewer psychiatric bed-days, higher rates of employment or education participation, and a trend toward reduced mortality. Compared to trial participants, real-world patients achieved equal or better outcomes [7].

*The UK Lambeth Early Outreach (LEO) *trial (London) evaluated Early intervention Services (EIS) over 18 months in a randomized design. EIS led to higher service engagement, lower rates of psychiatric readmission, and improved social functioning. Participants in the EIS group were less likely to relapse, had better quality of life, and greater satisfaction with care [8].

*The US Recovery After an Initial Schizophrenia Episode—Early Treatment Program (RAISE-ETP Trial)* was conducted across 34 community clinics in the USA [9]. This pragmatic RCT compared the NAVIGATE program (a manualized Early Intervention Service model) to standard community care. NAVIGATE participants had greater reductions in symptoms and improved quality of life, better functioning in work, school, and relationships, hospitalization rates were lower, especially when duration of untreated psychosis (DUP) was short, and patient satisfaction was significantly higher. The study emphasized the importance of early detection, shared decision-making, and coordinated team-based care.

Studies OPUS II in Denmark, Early Assessment Service for Young People with Psychosis (EASY) in Hong Kong and Program for Psychoses (PEPP) in Canada show that extending the duration of early intervention services (e.g., 5 years) can preserve or enhance early gains, particularly in social recovery and long-term integration.

Early intervention services have revolutionized the treatment landscape for psychosis, offering hope for improved outcomes in a traditionally high-burden condition. To maximize their impact, health systems must ensure high-fidelity implementation, provide adequate staffing and training, and support continuity of care beyond 2 years.

## The welcoming approach

The first consultation with a young person experiencing psychosis is a pivotal moment. Often, the individual has endured a long and difficult journey before arriving—grappling with symptoms, multiple consultations, and high personal barriers. It is essential to acknowledge this by treating the patient as a long-awaited guest, creating a safe, respectful, and welcoming environment from the start. Trust takes time. Patients may initially withhold or alter information due to fear, shame, or confusion. It is crucial not to judge or label this as deceit, but to understand it as part of building trust. With time, patients often reveal highly sensitive experiences, such as trauma, substance use, or past criminal behavior. A welcoming approach rejects punitive models like “three strikes and you’re out.” Those who miss appointments often need support the most. Persistent outreach and alliance-building—sometimes involving relatives—are vital. In rare cases, compulsory admission may be necessary, but should never be framed as punishment; rather, it is a last resort to ensure safety and care.

## Assertive Community Treatment

Guidelines from organizations like the APA, NICE, and EPA emphasize the importance of holistic, individualized treatment for people with severe mental illness. Assertive Community Treatment (ACT) and Intensive Case Management (ICM) are key models that provide intensive, around-the-clock multidisciplinary support, especially for individuals who struggle to engage with traditional services or experience frequent hospitalizations. Integrated mental health and addiction services are particularly important for those with co-occurring substance use and homelessness.

ACT is a specialized form of intensive case management focused on maintaining engagement, reducing hospital stays, and improving outcomes such as social functioning and quality of life. A defining feature of ACT is its community-based approach: rather than relying on institutional settings, care is delivered directly in the patient’s environment. Assertive outreach is central to this model, with teams proactively visiting patients at home to establish and maintain contact.

To ensure intensive and personalized care, ACT teams maintain small caseloads—typically no more than 15 patients per case manager. This allows for comprehensive, integrated support, including medication management, practical assistance, and help with social inclusion, such as accompanying patients to community spaces. Most services are provided directly by the team to promote continuity of care, and responsibilities are shared among team members to ensure consistent support.

## Vocational and educational support

Employment and education play a vital role in recovery for people with severe mental illness, offering not only financial stability but also a sense of purpose and improved wellbeing. Despite this, unemployment remains high in this group, with significant personal and societal costs.

To address this, the “Individual Placement and Support” (IPS) model was developed. Unlike traditional vocational rehabilitation, IPS focuses on rapid access to competitive employment or education based on individual preferences, integrates with mental health services, and includes ongoing job support and benefit counselling.

Several meta-analyses have shown beneficial effects of the IPS model [11]. A Danish randomized trial involving 720 participants compared IPS, an enhanced version with cognitive and social skills training (IPSE), and standard services. Over 18 months, participants in the IPSE group spent an average of 488 h in competitive employment or education, significantly more than the 341 h in the standard services group. The IPS group also improved (411 h), though the difference was smaller. While vocational outcomes between IPS and IPSE did not significantly differ, both IPS groups reported higher satisfaction with services. Nonvocational outcomes were similar across all groups [12].

## Housing First

A particularly vulnerable subgroup includes individuals with severe mental illness who are homeless or unstably housed. Traditional service models often fail to engage these individuals, who face complex barriers including substance use, trauma, and legal issues.

Evidence supports the effectiveness of the Housing First approach [13], which prioritizes immediate access to stable housing without preconditions. Once housed, individuals receive coordinated mental health, substance use, and social support. This model has been shown to reduce hospitalizations, improve housing stability, and enhance quality of life. The housing first concept has been tried out in the “chez soi” program in Canada, and it is implemented in many other countries.

## Avatar treatment of auditory hallucinations

Severe psychotic symptoms, such as persistent auditory hallucinations, can significantly impair daily functioning and social interaction. For some individuals, these symptoms make it difficult to leave home or use public transport. Auditory verbal hallucinations—often derogatory or threatening—affect 60–70% of people with schizophrenia. While medication helps many, around 25% continue to experience these symptoms despite treatment.

Recent therapies focus on the relational nature of these hallucinations, which are often perceived as voices with distinct identities and intentions. The hearer typically experiences a power imbalance, feeling dominated and powerless in relation to the voice—mirroring broader social dynamics. In response, new interpersonal therapies aim to address this imbalance by focusing on the relationship between the person and the voice.

Several clinical trials [14, 15] have shown that using virtual representations of hallucinatory content can effectively reduce distress and improve outcomes. This approach is particularly helpful for individuals with treatment-resistant schizophrenia but can also be used alongside medication in early stage psychosis.

## Familial risk and early intervention

Children of parents with severe mental illness—particularly schizophrenia and bipolar disorder—are at significantly increased risk of developing psychiatric conditions themselves. Studying these children provides a unique window into the early processes that precede the onset of mental disorders, allowing for the identification of modifiable risk factors and the development of targeted preventive interventions.

Since early signs of disorders like schizophrenia and bipolar disorder are uncommon in the general population, research in high-risk cohorts offers a more focused approach to understanding the development of psychopathology. These studies can reveal critical insights into symptom emergence, neurocognitive development, and structural and functional brain changes over time. Moreover, they help identify early environmental and psychological risk factors such as trauma, parenting difficulties, and early cognitive or emotional deviations.

The *Danish High Risk and Resilience Study—VIA 7* is a landmark example. This nationally representative cohort includes 522 seven-year-old children born to parents with schizophrenia, bipolar disorder, or no severe mental illness. The cohort has been followed longitudinally and reassessed at ages 11, 15, and now 19, allowing researchers to track developmental trajectories and identify early markers of resilience or vulnerability. The study provides a robust framework for early detection and intervention strategies aimed at altering the long-term course of severe mental illness.

## Physical health

For people with mental illness, the excess risk of dying from physical diseases is high and has not declined in recent years. In fact, individuals with mental illness are often diagnosed with both chronic and acute physical conditions at a younger age than the general population.

Several structural barriers contribute to this disparity. These include limited availability of services in rural areas, transportation difficulties, physical and mental disabilities, and challenges faced by people in supported accommodations, shelters, or correctional facilities. Despite legislation promoting equal access to healthcare in Denmark, out-of-pocket expenses remain for services such as dental care, physiotherapy, psychological treatment, and parts of medication costs. Social inequality also affects access to supplementary health insurance.

Individual factors such as health literacy and support networks further influence access to healthcare. Effective treatment for severe mental illness requires cross-sector coordination. In many in- and outpatient setting, general practitioners (GPs) or specialists in internal medicine are employed to reduce underdiagnosis and undertreatment.

While GPs are often the first point of contact, they may lack the necessary resources to manage complex psychiatric needs alone. Shared care models, where GPs work closely with psychiatrists, psychologists, and social workers, offer a more comprehensive approach. Tools like shared health records, joint case conferences, and clear referral pathways are essential to support collaboration. Integrating psychiatric consultations into general practice settings can also enhance access and promote early intervention.

## Pharmacological treatment

Antipsychotic medication remains a cornerstone in the treatment of schizophrenia. It plays a crucial role in reducing positive symptoms such as hallucinations and delusions, preventing relapse, and enabling recovery. For individuals who do not respond sufficiently to standard antipsychotic treatments, clozapine is the recommended option due to its superior efficacy in treatment-resistant cases.

To address challenges with adherence, especially in individuals with recurring relapses, long-acting injectable formulations can be particularly effective. These ensure a more stable delivery of medication and reduce the risk of unintentional discontinuation.

However, antipsychotic treatment must always be tailored to the individual. While continuous pharmacological treatment is necessary for many, some people with schizophrenia may, over time, successfully reduce or discontinue antipsychotic medication without relapse. This process—referred to as *deprescribing*—should be approached cautiously, gradually, and under close clinical supervision. Success is more likely in individuals with sustained remission, strong support systems, and low risk factors for relapse.

Acknowledging that long-term needs vary across individuals, treatment plans should balance the benefits of medication with side effects, patient preferences, and functional outcomes. A collaborative and flexible approach is essential, combining pharmacological strategies with psychosocial support to promote long-term recovery and autonomy.

## Crisis response and acute outreach services

People with severe mental disorders often experience crises that require urgent care. Standard emergency departments may not be equipped to handle psychiatric emergencies effectively, leading to delayed or inappropriate care.

In response, regions like the Capital Region of Denmark have developed Acute Psychiatric Outreach teams. These mobile units include a psychiatrist and a trained ambulance driver and are equipped to respond directly to people in their homes or community settings. They can assess patients, initiate treatment, and coordinate referrals. The service also works closely with the police in potentially dangerous situations to ensure safety and facilitate care.

Evaluations of the outreach model show that it provides timely, humane, and effective crisis intervention. It reduces unnecessary hospital admissions and supports voluntary engagement with mental health services. This model represents a scalable solution for crisis response that balances patient needs with public safety concerns.

## Conclusion

Improving outcomes in severe mental disorders demands a multi-pronged, coordinated approach. Early intervention, acute crisis outreach, housing and social support for vulnerable populations, integrated care models, and person-centered, evidence-based treatment must all be part of a comprehensive strategy. Denmark, through programs like OPUS and Acute Psychiatric Outreach, provides valuable models that can inform international practice. Sustained investment, policy support, and cross-sector collaboration are essential to realize the full potential of these services.

By focusing on services that are not only clinically effective but also cost-efficient and humane, we can offer individuals with severe mental disorders a better chance at recovery, dignity, and participation in society. All these elements must be delivered in a recovery-oriented framework that respects the person’s preferences, strengths, and goals.
